# Supplementary material for: Phenolic Profiles of Red Wine Relate to Vascular Endothelial Benefits Mediated by SIRT1 and SIRT6
Source: Int J Mol Sci. 2021 May 26;22(11):5677. doi: 10.3390/ijms22115677 (PMC8198779; doi:10.3390/ijms22115677)
Supplement: Supplementary file 1 [file ijms-22-05677-s001.zip › ijms-1233081-supplementary.pdf]

Phenolic Profiles of Red Wine Relate to Vascular Endothelial Benefits Mediated by SIRT1 and SIRT6

SUPPLEMENTARY FIGURES & TABLES

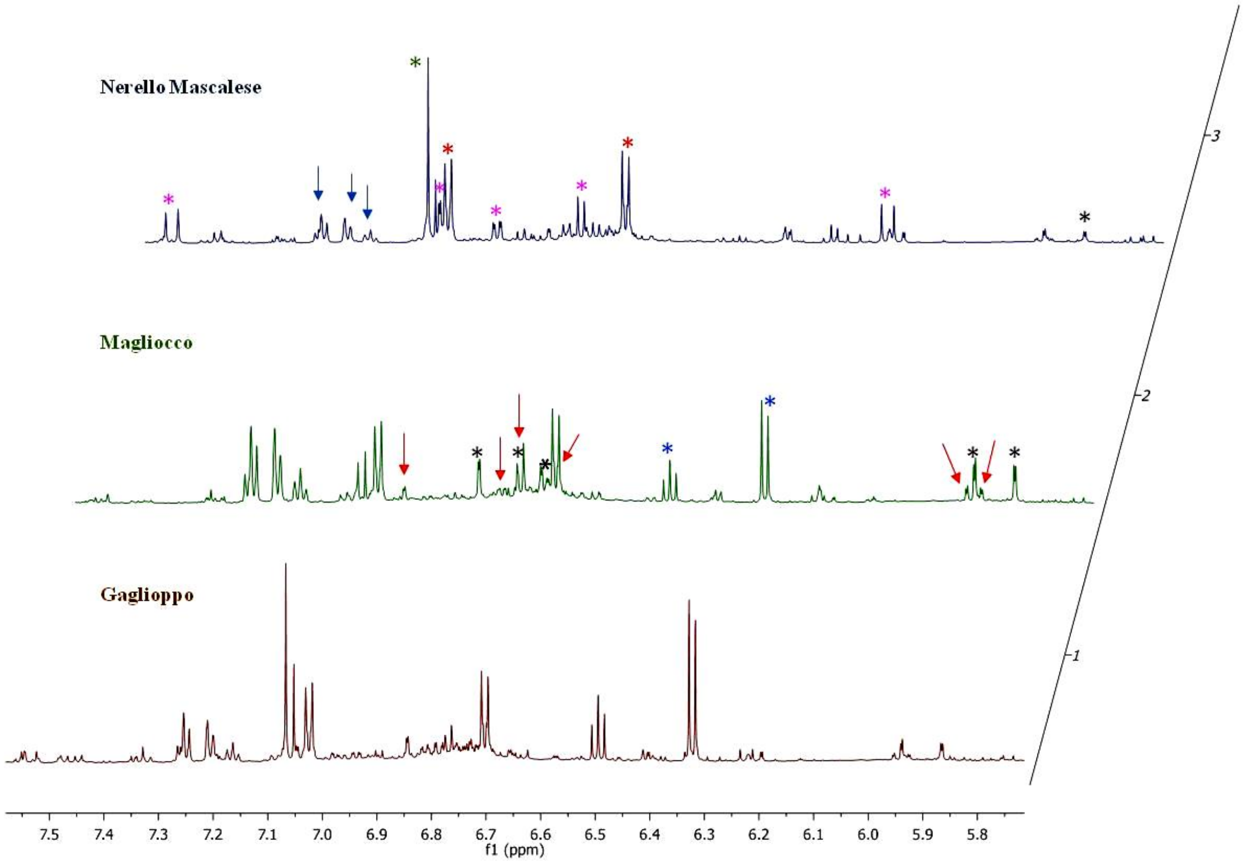

Supplementary Figure S1. Aromatic region of ^{1}H NMR spectra in CD_3OD of the ethyl acetate extracts of the red wines . Asterisks and arrows identify major wine polyphenols: ethyl caffeate (pink asterisks), 2-phenylethanol (blue arrows), gallic acid (green asterisk), tyrosol (red asterisks), catechin (black asterisks), epicatechin (red arrows), and pyrogallol (blue asterisks).

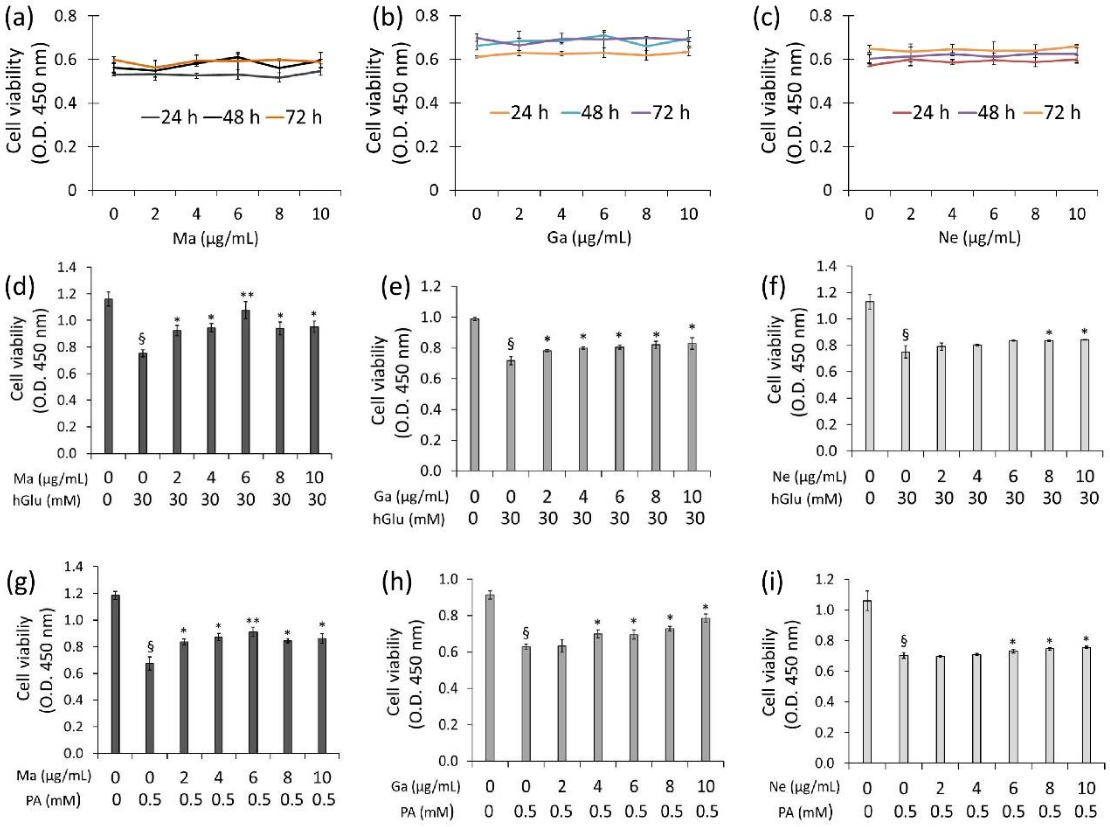

Supplementary Figure S2. Cytoprotective effects of wines during insulin-resistance and hyperglicaemia. (a-c) Dose-dependent effects of Magliocco (Ma), Gaglioppo (Ga) and Nerello (Ne) wines (up to 10 µg/mL) on endothelial cells viability after incubation for 24 h, 48 h, and 72 h. Effect of co-treatment of Ma, Ga or Ne (6 µg/mL) with (d-f) high-glucose (hGlu) (30 mM) or (g-i) palmitic acid (PA) (0.5 mM) for 48 h. Control cells were treated with corresponding volumes of Hanks' balanced salt solution (HBSS)-10 mM Hepes. §$p < 0.01$ vs Ctr, *$p < 0.05$ vs hGlu or PA, **$p < 0.01$ vs hGlu or PA.

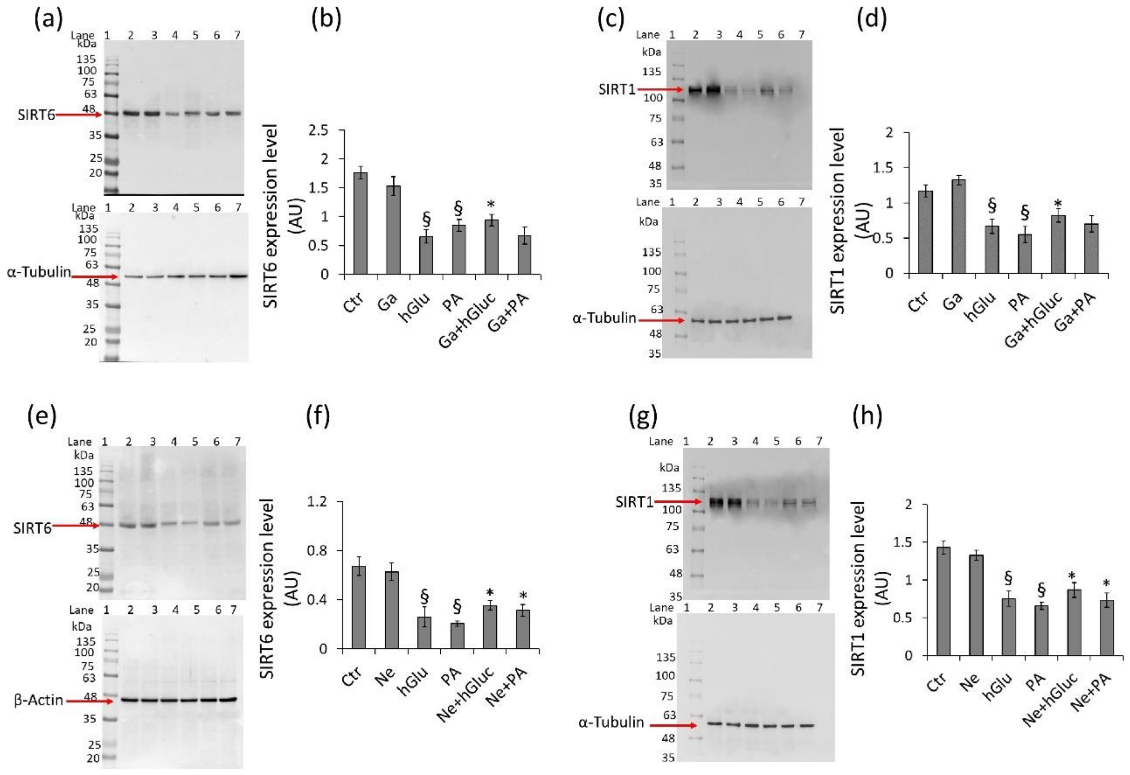

Supplementary Figure S3. SIRT1 and SIRT6 modulation by Gaglioppo and Nerello. (a,b) Western blot analysis of SIRT6 and (c, d) SIRT1 expression levels in endothelial cells after treatment with Ga before exposure to hGlu (30 mM) or PA (0.5 mM) for 48 h. Lane 1 = protein ladder molecular weight markers, lane 2 = Ctr, lane 3 = Ga, lane 4 = hGlu, lane 5 = PA, lane 6 = Ga+hGlu, lane 7 = Ga+PA. (e,f) Western blot analysis of SIRT6 and (g, h) SIRT1 expression levels in EC after treatment with Ne before incubation with hGlu (30 mM) or PA (0.5 mM) for 48 h. Control cells were treated with corresponding volumes of Hanks' balanced salt solution (HBSS)-10 mM Hepes. Lane 1 = protein ladder molecular weight markers, lane 2 = Ctr, lane 3 = Ne, lane 4 = hGlu, lane 5 = PA, lane 6 = Ne+hGlu, lane 7 = Ne+PA. The analysis of densitometric intensity was calculated with ImageJ software and expressed as arbitrary units (AU) ± SD of n = 3 replicates. α-Tubulin or β-actin was used as internal control. §$p < 0.01$ vs Ctr, *$p < 0.05$ vs hGlu or PA.

Supplementary Table S1. Base parameters of red wines determined by using official methods of analysis at bottling. Data are expressed as mean±SD of four replicates (two experimental replicates for two analytical replicates). For the chemical analysis two bottles for each treatment were analyzed. Alcohol, residuals sugar, titratable acidity, volatile acidity, and free and total sulfur dioxide were determined by the official method of analysis (OIV-MA-F1-07, RESOLUTION OIV-OENO 419A / 2011, www.oiv.int).

	**Gaglioppo**	**Magliocco**	**Nerello Mascalese**
EtOH (%)	13.05 ± 0.01	10.68 ± 0.02	11.47 ± 0.02
pH	3.55 ± 0.01	3.84 ± 0.08	3.77 ± 0.01
Free SO_2 (mg/L)	11.1 ± 0.6	7.15 ± 0.05	2.4 ± 0.01
Total SO_2 (mg/L)	61.60 ± 0.46	26.4 ± 0.16	16.75 ± 1.9
Titratable acidity (mg/L)	5.57 ± 0.1	4.65 ± 0.01	4.2 ± 0.05
Volatile acidity (mg/L)	0.93 ± 0.02	0.61 ± 0.01	0.64 ± 0.015
Residual sugars (mg/L)	1.92 ± 0.09	1.39 ± 0.01	1.7 ± 0.14
